# Nutrient enhancement of allelopathic effects of exotic invasive on native plant species

**DOI:** 10.1371/journal.pone.0206165

**Published:** 2019-01-23

**Authors:** Tao Xiao, Hua Yu, Yao-Bin Song, Yue-Ping Jiang, Bo Zeng, Ming Dong

**Affiliations:** 1 Key Laboratory of Eco-environments in Three Gorges Reservoir Region (Ministry of Education), Chongqing Key Laboratory of Plant Ecology and Resources in Three Gorges Reservoir Region, School of Life Sciences, Southwest University, Chongqing, China; 2 Key Laboratory of Hangzhou City for Ecosystem Protection and Restoration, College of Life and Environmental Sciences, Hangzhou Normal University, Hangzhou, China; 3 Hangzhou Xixi National Wetland Park Research Center for Ecological Sciences, Hangzhou, China; Shandong University, CHINA

## Abstract

Many ecosystems may suffer from both nutrient enrichment and exotic plant invasions simultaneously. Much has been known that nutrient inputs can promote growth and expansion of exotic invasive plants in wetlands, and that allelopathic effects of the exotic invasive plants can inhibit the growth of coexisting native plants, contributing to their invasion success. Thus, we hypothesized that allelopathic effects of exotics on natives in invaded ecosystems can be enhanced by nutrient enrichment. To test this hypothesis, we conducted two greenhouse hydroponic experiments. One is the monoculture experiment in which a widespread exotic invasive perennial *Alternanthera philoxeroides* and a native perennial *Ludwigia peploides* subsp. *stipulacea* in monoculture were subjected to five levels of nutrient supply. The other is the mixture experiment in which the two species in mixture were subjected to five levels of nutrient supply, each with and without activated carbon addition. Both *A*. *philoxeroides* and *L*. *peploides* grew better under higher level of nutrient availability in monoculture experiment. In the mixture experiment, *A*. *philoxeroides* formed less total and root biomass while *L*. *peploides* formed more in response to activated carbon addition and all of the responses had larger degree at higher level of nutrient availability, indicating *A*. *philoxeroides* had significant allelopathic effects on *L*. *peploides* and the effects was significantly enhanced by nutrient enrichment. Such results support our hypothesis and reveal a novel mechanism for exotic plant invasion in eutrophicated and invaded wetlands, *i*.*e*. nutrient enhancement of allelopathic effects of exotics on natives.

## Introduction

Wetlands can actually or potentially offer many ecosystem services to human society [1, 2]. However, human-induced municipal sewage discharging, agricultural fertilization [3, 4], and/or atmospheric nitrogen deposition [5] may increase nitrogen and/or phosphorous loading to wetlands, causing water eutrophication of the ecosystems. This is particularly true in urban and suburban wetlands [6, 7]. In fact, as a consequence of global environmental change, around the world eutrophication has been leading to wetland ecosystem degradation [8–11], which is often characterized by biodiversity losing and productivity decreasing [12–14].

Wetlands in economically developed regions often suffered not only eutrophication but also biological invasion [6, 15]. Previous studies found wetlands become more susceptible to exotic plant invasions after suffering from human disturbances like eutrophication due to nutrient accumulation [12, 16, 17]. Exotic invasive plants can alter ecosystem processes profoundly in wetlands [15, 18, 19]. Recent studies found nutrient inputs could promote expansion of exotic invasive plants in wetlands [12, 20], which can also shift growth and interactions between exotic invasive and native species, endowing the exotic invasive plants with competition advantage [21–23] and consequently with a higher invasiveness. Thus, understanding how wetland ecosystems respond to nutrient accumulation and plant invasion can greatly help ecosystem management and governance of wetlands [6, 24].

Allelopathy between plants refers to the effect of toxic metabolites produced and released by a plant species on the growth of another [25, 26]. Novel weapon hypothesis claimed that allelopathic effects of the exotic on the native plant species substantially contribute to the invasion success through inhibiting growth of the native [26–28]. So far, we have known that plant invasion can be promoted by nutrient enrichment [12, 29, 30] and by allelopathic effects of the exotics on the natives [27, 28, 31–33], while we have not known whether nutrient enrichment can enhance allelopathic effects of the exotics on the natives, being among the mechanisms underlying the promotion. Thus, we propose a new hypothesis that nutrient enrichment can enhance the allelopathic effects of the exotics on the natives.

To test this hypothesis, we conducted two greenhouse hydroponic experiments. One is a monoculture experiment in which *Alternanthera philoxeroides*, an exotic clonal perennial widely invading in China, and *Ludwigia peploides* subsp. *stipulacea*, a native clonal perennial, were grown in monoculture and subjected to five levels of nutrient supply. The other is a mixture experiment in which the two plant species were grown in mixture and subjected to five levels of nutrient supply, each with and without activated carbon addition. Previous studies found that nutrient availability could promote the growth and competitive ability of *A*. *philoxeroides* [23, 34]. Therefore, based on the new hypothesis, we predict: 1) the exotics will grow better under the higher level of nutrient availability; 2) activated carbon addition will decrease the allelopathy increasing with the growth of the exotics; 3) allelopathic effect of the exotics on the natives will be larger under higher level of nutrient availability.

## Materials and methods

### Species and plant materials

*Alternanthera philoxeroides* (Mart.) Griseb. (Amaranthaceae), a perennial stoloniferous clonal plant native to South America, is a serious exotic invasive species spread to Australia, New Zealand, USA, Thailand and China. It is amphibious so that it is able to grow both in wetland and terrestrial habitat [35]. *A*. *philoxeroides* has extremely low genetic diversity in China [36, 37], and mainly propagates through clonal growth by formation of stolon, rhizome and tuber [35]. This species can produce aqueous and degradable allelochemicals to inhibit co-occurring native species, especially for aquatic ecotype [38].

*Ludwigia peploides* subsp. *stipulacea* (syn. *Jussiaea repens*; Onagraceae; hereafter abbreviate as *L*. *peploides*), is a perennial stoloniferous clonal plant growing in wetland habitats, such as bank of canals, ponds and paddy fields [39]. It is a native species in China and mainly distributed in Zhejiang Province, Fujian Province and the East of Guangdong Province. The two species usually coexist in many wetlands from aquatic to aquatic-terrestrial ecotones in South China [39, 40]. Previous studies found *A*. *philoxeroides* have a competitive advantage over *L*. *peploides* in heterogeneous environments due to the ability of clonal integration [39].

Plant materials of *A*. *philoxeroides* and *L*. *peploides* were collected from the Xixi National Wetland Park (30°14′-30°16′N, 120°02′-120°05′E) located in Hangzhou City, Zhejiang Province, China, with the approval by Administration of Xixi National Wetland Park. This study did not involve any endangered or protected species. To avoid sampling the same genotypes, we collected materials of each species from at least five locations at least 20 m apart. The plant materials were propagated in a greenhouse at Hangzhou Normal University, China. After 2 weeks of recovery growth, tip cuttings of *A*. *philoxeroides* and *L*. *peploides* respectively were selected and planted into plastic containers with Hoagland solution for continued culture.

### Experimental design

#### Monoculture experiment

On September 10, 2017, 25 cuttings with 10 cm length and similar size of *A*. *philoxeroides* and *L*. *peploides* were grown in total 50 (H × L × W: 12.5cm × 40cm × 30cm, 15 L totally) plastic containers in the greenhouse, separately. We set five nutrient levels (Table 1; N1 to N5) with different nitrogen and phosphorous concentrations adjusted by NH_4_NO_3_ and Na_2_HPO_4_ solution, referring to eutrophication situation of the Yangtze Delta Region [41] where the plant materials were collected. Nitrogen to phosphorous ratio (N:P) of the solution was the same (20:1) for all five nutrient levels, and kept consistent during the experiments to avoid the potential confounding effects of N:P on interspecific interactions [23]. The concentrations of other essential elements for plant growth in the nutrient solution were referred to Hoagland solution. The 25 containers of each species were randomly subjected to the five nutrient treatments, each with five replications. Totally, there were 2 species × 5 nutrient treatments × 5 replications.

**Table 1:** Nitrogen and phosphorus dose of different nutrient levels in the experiments.

Nutrient level	NH_4_NO_3_ (mg)	Na_2_HPO_4_ (mg)	[N] (mg L^-1^)	[P] (mg L^-1^)
N1	1.43	0.11	0.2	0.01
N2	7.14	0.57	1.0	0.05
N3	14.29	1.15	2.0	0.10
N4	21.43	1.72	3.0	0.15
N5	28.57	2.29	4.0	0.20

#### Mixture experiment

At the same time, 50 cuttings with 10 cm length and similar size for each of the species were randomly chosen and grown in the way in which one cutting of one of the two species together, with one cutting of the other were in each of in total 50 plastic containers (the same size as in monoculture experiment) put randomly in the greenhouse. The 50 containers of each species were randomly subjected into the five nutrient treatments (the same as in monoculture experiment) with five replications, and half of them were assigned into activated carbon addition (with the dosage of 2%, 250 g per container) treatment to neutralize potential allelopathic effects [42, 43]. Totally, there were 5 nutrient treatments × 5 replications.

The two experiments lasted two months (from September 10 to November 10, 2017) in the greenhouse. Nutrient solution and activated carbon were replenished every 7 days. During the experiments, containers were supplied with deionized water once a day and the water level in the containers was kept. Containers with different treatments were randomly arranged in the greenhouse to avoid potential confounding effects of local environmental conditions. Additionally, all containers were repositioned every week to avoid the effects of possible environmental patchiness within the greenhouse.

### Harvest and measurements

At harvest, we separated each plant into leaf, stolon and root. Then all materials of the different plant parts were oven-dried at 60°C for 48 h before they were weighed respectively.

### Data analysis

For the monoculture experiment, two-way ANOVA was performed to test the effects of nutrient level and species identity on total biomass, root biomass, stolon biomass and leaf biomass accumulation. For the mixture experiment, three-way ANOVA were applied to examine the effects of nutrient availability, species identity and activated carbon addition on total biomass, root biomass, stolon biomass and leaf biomass accumulation. When ANOVA showed significant differences, we conducted LSD tests to make multiple comparisons among treatments within species. Data were transformed to meet the assumption of ANOVA when needed. All statistical analyses were conducted using SPSS 22.0 (SPSS, Chicago, IL, USA). An effect was considered significant if *P* < 0.05.

## Results

### Plant performance in monoculture experiment

**Fig 1:**
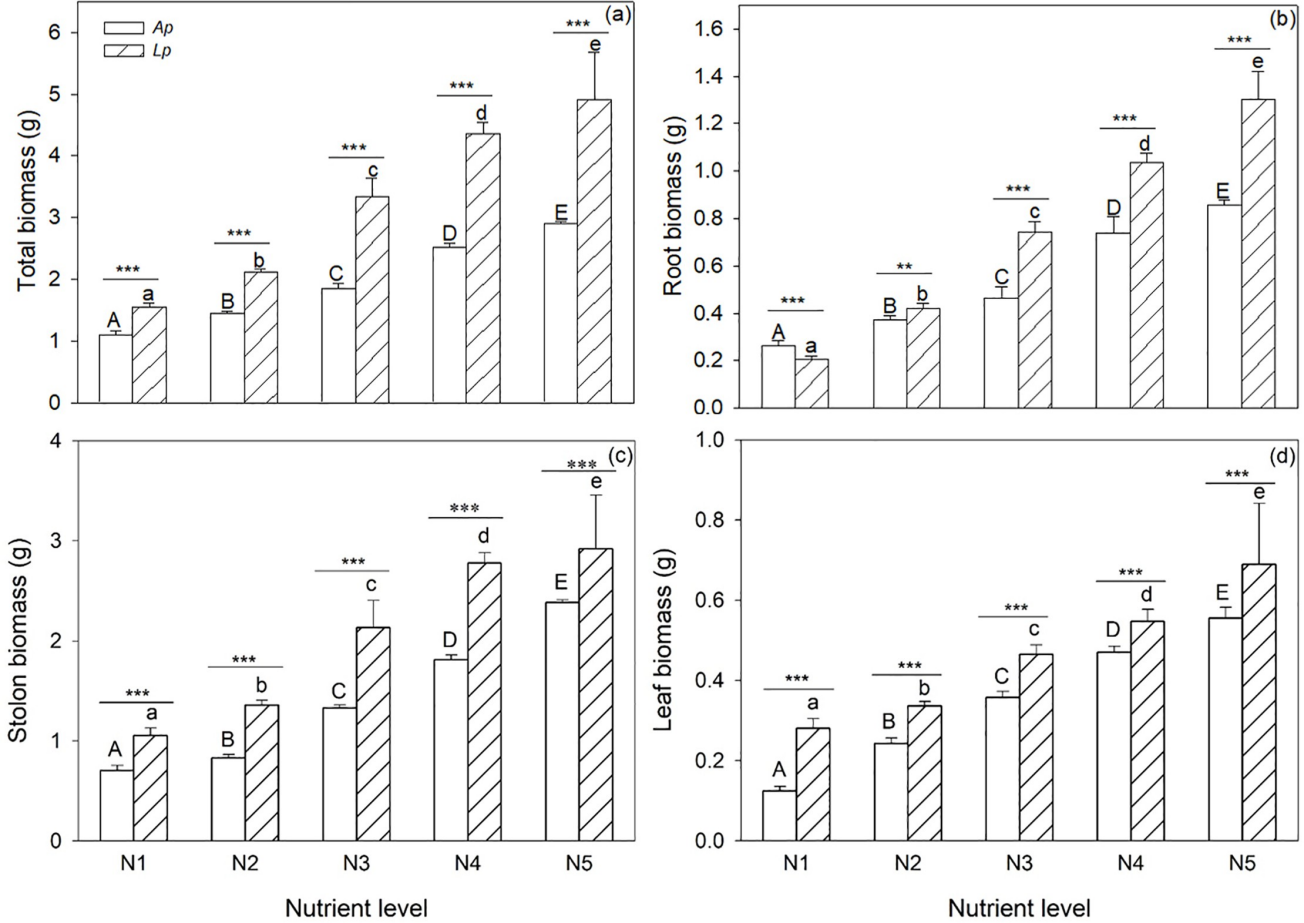
Total biomass (a), root biomass (b), stolon biomass (c) and leaf biomass (d) of *Alternanthera philoxeroides* (*Ap*) and *Ludwigia peploides* (*Lp*) under different nutrient levels in monoculture experiment. Values are presented as means + SE (n = 5). Bars with different letters are significantly different at *P* = 0.05 for each species. The overlined two bars with *, ** and *** are significantly different at *P* = 0.05, *P* = 0.01 and *P* = 0.001, respectively.

Species identify, nutrient availability and their interaction all significantly affected total biomass, root biomass, stolon biomass and leaf biomass of plants in the monoculture experiment (Table 2). Total biomass, root biomass, stolon biomass and leaf biomass of *A*. *philoxeroides* and *L*. *peploides* increased significantly with the elevation of nutrient supply (Fig 1; Table 2). Under the same nutrient level, total biomass, root biomass, stolon biomass and leaf biomass of *A*. *philoxeroides* were significantly smaller than those of *L*. *peploides* (but see root biomass under N1 nutrient level) (Fig 1; Table 2), and such interspecific differences in total biomass, root biomass, stolon biomass and leaf biomass significantly enlarged with the elevation of nutrient level (Fig 1; Table 2).

**Table 2:** Effects of species identity (S) and nutrient availability (N) on total biomass, root biomass, stolon biomass and leaf biomass of plants in the monoculture experiment with *Alternanthera philoxeroides* or *Ludwigia peploides*.

	Source	d.f.	F	*P*
Total biomass	S	1,38	254.543	<0.001
	N	4,38	142.562	<0.001
	S × N	4,38	14.685	<0.001
Root biomass	S	1,38	178.767	<0.001
	N	4,38	410.447	<0.001
	S × N	4,38	35.216	<0.001
Stolon biomass	S	1,38	276.344	<0.001
	N	4,38	76.325	<0.001
	S × N	4,38	15.140	<0.001
Leaf biomass	S	1,38	55.518	<0.001
	N	4,38	95.777	<0.001
	S × N	4,38	0.833	0.513

### Plant performance in mixture experiment

**Fig 2:**
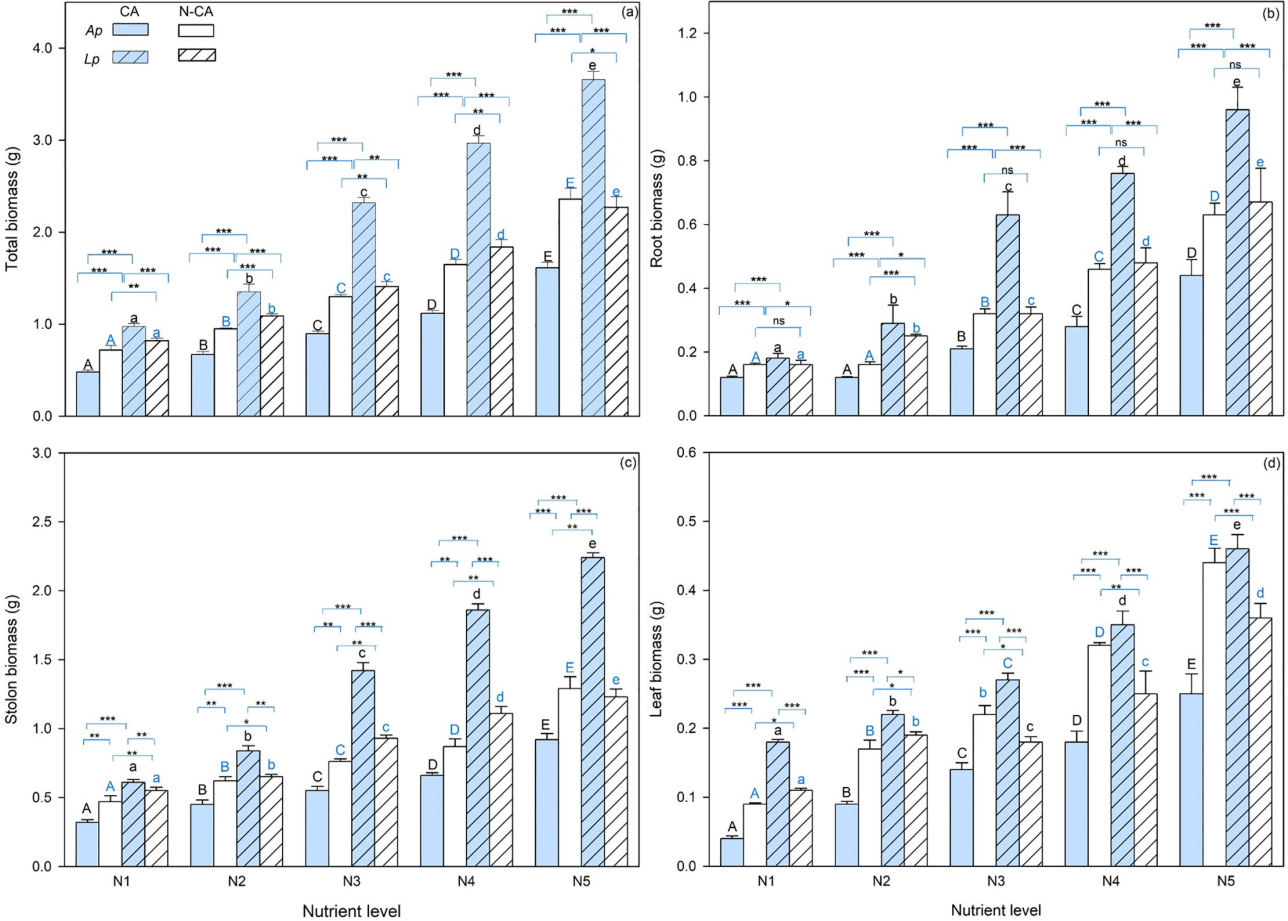
Total biomass (a), root biomass (b) stolon biomass (c) and leaf biomass (d) of *Alternanthera philoxeroides* (*Ap*) and *Ludwigia peploides* (*Lp*) under different nutrient levels in the mixture experiment with (CA) and without (N-CA) activated carbon addition. Values are presented as means + SE (n = 5). The bars with different letters are significantly different at *P* = 0.05 for each species with or without active carbon addition. The overlined two bars with *, ** and *** are significantly different at *P* = 0.05, *P* = 0.01 and *P* = 0.001, respectively. The overlined two bars with *ns* are not different at *P* = 0.05.

Total biomass, root biomass, stolon biomass and leaf biomass of plants in the mixture experiment were significantly affected by activated carbon addition, species identity, nutrient level and their interactions (Table 3). In response to activated carbon addition, total biomass, root biomass, stolon biomass and leaf biomass significantly decreased in *A*. *philoxeroides* while increased in *L*. *peploides* (Fig 2). More interestingly, the plants grew under higher nutrient level, the increase and the decrease were significantly larger, as shown in Fig 2, and indicated by the significant interaction effects of C × S × N at *P* = 0.001 in Table 3.

**Table 3:** Effects of activated carbon addition (C), species identity (S) and nutrient availability (N) on total biomass, root biomass, stolon biomass and leaf biomass of plants in the mixture experiment with *Alternanthera philoxeroides* and *Ludwigia peploides*.

	Source	d.f.	F	*P*
Total biomass	C	1,80	173.652	<0.001
	S	1,80	3067.657	<0.001
	N	4,80	2417.841	<0.001
	C × S	1,80	2319.590	<0.001
	S × N	4,80	136.166	<0.001
	C × N	4,80	38.838	<0.001
	C × S × N	4,80	174.340	<0.001
Root biomass	C	1,80	22.567	<0.001
	S	1,80	477.482	<0.001
	N	4,80	537.932	<0.001
	C × S	1,80	328.398	<0.001
	S × N	4,80	28.864	<0.001
	C × N	4,80	5.727	<0.001
	C × S × N	4,80	32.288	<0.001
Stolon biomass	C	1,80	298.889	<0.001
	S	1,80	3178.579	<0.001
	N	4,80	1717.657	<0.001
	C × S	1,80	2003.224	<0.001
	S × N	4,80	185.889	<0.001
	C × N	4,80	74.884	<0.001
	C × S × N	4,80	170.464	<0.001
Leaf biomass	C	1,80	19.695	<0.001
	S	1,80	420.743	<0.001
	N	4,80	1004.861	<0.001
	C × S	1,80	1019.630	<0.001
	S × N	4,80	8.168	<0.001
	C × N	4,80	14.524	<0.001
	C × S × N	4,80	34.514	<0.001

Under the same nutrient level, total biomass, root biomass, stolon biomass and leaf biomass of *A*. *philoxeroides* were significantly smaller than that of *L*. *peploides* (Fig 2; Table 3). And such interspecific differences in both total biomass, root biomass, stolon biomass and leaf biomass significantly enlarged with the elevation of nutrient level (Fig 2; Table 3).

## Discussion

Allelopathic effect was enlarged with the elevation of nutrient level. In both monoculture and mixture experiment, *A*. *philoxeroides* plants grew better in terms of total biomass, root biomass, stolon biomass and leaf biomass under higher level of nutrient availability. In the mixture experiment *A*. *philoxeroides* plants grew less in response to activated carbon addition and the degree of the responses was larger at higher level of nutrient availability, indicating that the species grew better in response to allelophathy with larger response degree at higher level of nutrient availability since activated carbon addition leads to the removal of allelochemicals [42–44]. Obviously, our results proved the predictions made in the introduction. Therefore the new hypothesis proposed in the introduction and based on which the predictions were made was supported, that is, nutrient enrichment can enhance the allelopathic effects of the exotics on the natives.

The correlation between nutrient enhancement and allelopathic effect gives new insights into the invasion mechanism of wetland invasive plants. There are many evidences for that exotic invasive plants can produce various allelochemicals, such as phenols, terpenoids and alkaloids, which would be beneficial to enhance their capacity of interspecific competition and to promote invasion [27, 31, 32]. Activated carbon is often used to manipulate the allelopathic interaction, because it strongly absorbs various allelochemicals while it has no affinity for hydrophilic molecules including most plant available nutrients [42–44]. Our results showed that activated carbon addition significantly reduced the total biomass, root biomass, stolon biomass and leaf biomass in *A*. *philoxeroides* while increased in *L*. *peploides* in the mixture experiment. Therefore, we inferred that *A*. *philoxeroides* obtained the net allelopathic effects in the mixture experiment, *i*.*e*., inhabitation of growth of *L*. *peploides* by allelochemicals released by *A*. *philoxeroides* was stronger than that of *A*. *philoxeroides* by *L*. *peploides* under the same nutrient level. More interestingly, the net eutrophic effects between *A*. *philoxeroides* and *L*. *peploides* tended to enlarge as the nutrient availability increased. This implicates that water eutrophication, together with allelopathic effects of exotic invasive plants, could alter the interspecific interactions between the exotic and the native plants, eventually excluding the remnant native species in invaded ecosystems and further lowering biodiversity of the communities. Our finding that nutrient enhancement of allelopathic effects of the exotics on the native supports to reveal a novel mechanism explaining the invasion success of the exotic plant in eutrophicated and invaded wetlands.

Associated with nutrient enrichment, allelopathic effects significantly affected the interaction pattern and growth status of both the native and the exotic invasive species. In monoculture experiment of this study, without regard to interspecific interaction due to natural enemies, resource shortage and allelochemical release, we found that both the native species and the exotic invasive species could significantly respond to nutrient enrichment and that the native species grew much better than the exotic invasive. This indicates that water eutrophication can stimulate the growth of wetland plants at species level, in consistent with many previous studies which also found that eutrophication increased the growth yields of terrestrial and wetland plants on individual scale [12, 17]. In mixture experiment, it is interesting that both the native *L*. *peploides* and the invasive *A*. *philoxeroides* had less biomass than they were in the monoculture experiment while the native *L*. *peploides* decreased biomass in a much higher rate. That is likely due to that the invasive *A*. *philoxeroides* exerted allelopathic effect on the native *L*. *peploides* to a much larger extend than the native did to the invasive. Native plants had some potential mechanisms, such as higher resource use efficiency or allelopathic effects, to resist invasions by exotic species [42]. However, after activated carbon treatment had alleviated the allelopathic effect on the native by the invasive to large extent, the total and root biomass of native greatly increased while that of the invasive dramatically decreased, and the effects was significantly enhanced by nutrient enrichment. It is in agreement with the findings of the previous research that *L*. *peploides* had larger individuals and more developed roots under eutrophic water, consequently a stronger nutrient absorption ability and a greater biomass accumulation rate [39]. And more interestingly, the growth advantage of the native over the exotics was bigger at higher level of nutrient availability. These suggest that native *L*. *peploides* might have a higher resource use efficiency than the invasive exotics when either they grow alone or together, and that is particularly true in eutrophicated environments. Thus, such a native plant species can be selected and applied to the restoration of invaded and/or eutrophicated wetland ecosystems, similar to those for restoring grassland invaded by spotted knapweed [45].

## Conclusions

Our results reveal a novel mechanism for the success of exotic plant invasion in eutrophicated and invaded wetland, *i*.*e*., nutrient enhancement of allelopathic effects of exotic on native species. This finding implicates that, due to the novel mechanism, eutrophication may considerably promote the invasion success of the exotic plants and accelerate their spatial expansion, particularly in wetlands. Future biological invasion managements should consider interspecific relationships and their interactions with the fast changing environments, especially eutrophication.
